# Maternal high-fat diet promotes calcified atherosclerotic plaque formation in adult offspring by enhancing transformation of VSMCs to osteochondrocytic-like phenotype

**DOI:** 10.1016/j.heliyon.2022.e10644

**Published:** 2022-09-15

**Authors:** Daisuke Miyawaki, Hiroyuki Yamada, Makoto Saburi, Naotoshi Wada, Shinichiro Motoyama, Takeshi Sugimoto, Hiroshi Kubota, Noriyuki Wakana, Daisuke Kami, Takehiro Ogata, Satoaki Matoba

**Affiliations:** a Department of Cardiovascular Medicine, Graduate School of Medical Science, Kyoto Prefectural University of Medicine, Kyoto, Japan; b Department of Regenerative Medicine, Graduate School of Medical Science, Kyoto Prefectural University of Medicine, Kyoto, Japan; c Department of Pathology and Cell Regulation, Graduate School of Medical Science, Kyoto Prefectural University of Medicine, Kyoto, Japan

**Keywords:** Maternal high-fat diet, Atherosclerosis, Cardiovascular calcification, Interleukin-1β

## Abstract

**Aim:**

Maternal high-fat diet (HFD) is associated with the development of cardiovascular disease (CVD) in adult offspring. Atherosclerotic vascular calcification is well documented in patients with CVD. We examined the effect of maternal HFD on calcified plaque formation.

**Methods and results:**

Seven-week-old female apo-E^−/−^ mice (C57BL6/J) were nourished either an HFD or a normal diet (ND) a week before mating, and during gestation and lactation. Offspring of both the groups were fed a high-cholesterol diet (HCD) from 8 weeks of age. Osteogenic activity of the thoracic aorta, assessed using an ex vivo imaging system, was significantly increased after 3 months of HCD in male offspring of HFD-fed dams (O-HFD) as compared with those of ND-fed dams (O-ND). Alizarin-red-positive area in the aortic root was significantly increased after 6 months of HCD in male O-HFD as compared to that of O-ND. Plaque size and Oil Red O-positive staining were comparable between the two groups. Primary cultured vascular smooth muscle cells (VSMCs) of the thoracic aorta were treated with phosphate and interleukinL-1β (IL-1β) to transform them into an osteochondrocytic-like phenotype. Intracellular calcium content and alkaline phosphatase activity were markedly higher in the VSMCs of O-HFD than in O-ND. IL-1β concentration in the supernatant of bone marrow-derived macrophages was markedly higher in O-HFD than in O-ND.

**Conclusion:**

Our findings indicate that maternal HFD accelerates the expansion of atherogenic calcification independent of plaque progression. In vitro phosphate- and IL-1β-induced osteochondrocytic transformation of VSMCs was augmented in O-HFD. Inhibition of VSMCs, skewing toward osteochondrocytic-like cells, might be a potential therapeutic strategy for preventing maternal HFD-associated CVD development.

## 1 Introduction

Maternal overfeeding and obesity during gestation and lactation are known to increase the risk of cardiovascular diseases (CVD) and cardiometabolic abnormalities during the lifetime of the offspring [1, 2, 3, 4]. We found that maternal high-fat diet (HFD) intake accelerates atherosclerosis development in offspring via an amplified macrophage-mediated inflammatory effects [5]. We have also demonstrated that maternal HFD augments diet-induced insulin resistance (IR) in offspring by promoting inflammasome activation and increasing the expression of macrophage interleukin-1β (IL-1β) [6].

Atherosclerotic cardiovascular calcification has long been recognized as a predictor of incident CVD and is strongly correlated with major adverse cardiovascular events [7, 8], particularly in patients with diabetes mellitus and metabolic syndrome [9, 10, 11]. Intensive studies have elucidated the underlying mechanisms of cardiovascular calcification [12, 13, 14], in which the transdifferentiation of vascular smooth muscle cells (VSMCs) plays a central role [15, 16, 17, 18]. Of note, inflammatory macrophages have been reported to accelerate vascular calcification as well as atherosclerosis development by promoting VSMC transdifferentiation to the chondrogenic phenotype [19, 20, 21, 22]. Thus, we investigated whether maternal HFD promotes atherosclerotic vascular calcification.

Here, we investigated the effect of maternal HFD on atherosclerotic vascular calcification in the offspring. We show that maternal HFD increases osteogenic activity of the thoracic aorta and alizarin-red-positive area in the aortic root, independent of plaque progression. Furthermore, phosphate- and IL-1β-induced transdifferentiation of primary cultured VSMCs into osteochondrocytic-like cells was significantly increased by maternal HFD. These findings suggest that maternal HFD-associated CVD development is likely to be attributed, at least in part, to VSMCs skewing toward osteochondrocytic-like cells along with macrophage-derived IL-1β.

## 2 Materials and methods

### 2.1 Experimental animals

All experiments strictly adhered to Directive 2010/63/EU of the European Parliament and the Guidelines for NIH and Animal Experiments of the Kyoto Prefectural University of Medicine, following approval by the Institutional Animal Care and Use Committee of the Kyoto Prefectural University of Medicine (approval reference number: M2021-82).

ApoE deficient (apoE^−/−^) mice (B6.129P2-Apoe^tm1Unc^/J) were obtained from the Jackson Laboratory (Bar Harbor, ME, USA). Seven-week-old female mice were maintained either on a normal diet (ND: 12.0% fat, 28.9% protein, 59.1% carbohydrate; Oriental Yeast Co., Tokyo, Japan) or on a high-fat diet (HFD: 62% fat, 18.2% protein, and 19.6% carbohydrate; Oriental Yeast Co.) for one week before mating, as well as during gestation and lactation. Each dam delivered several pups and there was no discernible difference in litter size. Offspring of HFD-fed dams (O-HFD) and ND-fed dams (O-ND) were weaned at 5 weeks of age and fed an ND until the age of 8 weeks, then were switched to a high-cholesterol diet (HCD: 13.6% fat, 1.25% cholesterol; Oriental Yeast Co.) until the age of 5 or 8 months. One offspring per sex per dam was used in this experiment, and was not used for other experiments presented in this study. Animals were housed in a room maintained at 22 °C under a 12 h light/dark cycle and provided with drinking water *ad libitum*. After 3 or 6 months of HCD feeding, mice were euthanized by transcardial perfusion under anesthesia induced by isoflurane (2%; 0.2 mL/min). Timeline of experimental protocol is shown in Supplementary Figure 1.

### 2.2 Ex vivo osteogenic activity

OsteoSense 680 (2 nmol, PerkinElmer, Waltham, MA, USA) was administered to all animals via tail vein injection. OsteoSense 680 is a targeted fluorescence imaging tracer for early osteogenic activity in the vasculature through incorporation into hydroxyapatite. The thoracic aorta was harvested 24 h post–injection, followed immediately by ex vivo aorta imaging using an *in vivo* imaging system (IVIS, Lumina Serie III optical imaging platform, PerkinElmer Inc.) employing the red filter sets (excitation range, 680 nm; emission, 710 nm long pass), as previously described [23]. Regions of interest (ROI) encompassing the thoracic aortae were manually drawn and the resulting signal was calculated in units of scaled counts per second. We confirmed that the sizes of the ROIs drawn across animal samples were consistent.

### 2.3 Quantitative measurement of atherosclerotic lesions

Mice were euthanized after 3 or 6 months of HCD feeding, and atherosclerotic lesions were analyzed. Atherosclerotic lesions of the aortic root were examined at four or five locations, 50 μm apart. Imaging and analysis of Oil Red O-stained aortas were conducted using the ImageJ software v1.50i software (https://imagej.nih.gov/ij/index.html, accessed on 10 October 2021).

### 2.4 Alizarin red staining

Three serial sections (10 μm thick) were prepared from the aortic root and stained 40 mM fresh Alizarin Red solution (pH = 4.1–4.3, Alizarin-Red Staining Solution, Sigma-Aldrich, St. Louis, MO, USA) for 20 min at room temperature as previously described [24].

### 2.5 Immunohistochemical analysis

Three serial sections (10 μm thick) were prepared from the aortic root and immunohistochemically stained. For the immunological staining of Mac-3, Alexa Fluor® 647 anti-Mac-3 antibody (108512; BioLegend, California, USA) was used. Nuclei were labeled using 4', 6-diamidino-2-phenylindole (DAPI) (62248; Thermo Fisher Scientific), and sections were examined using an LSM 510 META confocal microscope (Carl Zeiss, Jena, Germany). Non-immune immunoglobulin Rat IgG1, κ Isotype Ctrl Antibody (400418; BioLegend) was used as a negative control. The percentages of Mac-3-positive stained areas were assessed in three sections from 4 O-ND and 4 O-HFD.

### 2.6 In vitro differentiation of VSMCs into osteochondrocytic-like cells

Aortic VSMCs were prepared from 8-week-old O-ND and O-HFD as described previously [25]. The media was stripped from the thoracic aorta under a dissection microscope, followed by incubation with 1 mg/mL collagenase type II (Worthington Biochemical Corporation. Lakewood, NJn USA) to remove residual endothelial and adventitial cells. Aortic media were then dispersed in a medium containing 1 mg/mL collagenase type II, 0.5 mg/mL elastase type III (Sigma-Aldrich), and 20% fetal bovine serum (FBS). Cell suspensions were centrifuged at 500 g for 5 min, and the cell pellets were resuspended in DMEM containing 100 U/mL penicillin, 100 Ag/mL streptomycin, and 20% FBS. Aortic VSMCs were seeded at a density of 1 × 10^5^ cells/mL for primary culture and split at 1:2 at confluency. The cells used for the experiments were obtained from the primary culture to passage 5. Subcultured SMCs were maintained in DMEM containing 1% penicillin/streptomycin and 20% FBS. To induce transformation, cells were cultured in DMEM containing 3% FBS and inorganic phosphate (Pi), as indicated below. VSMC calcification was induced by treatment with calcification media supplemented with NaH_2_PO_4_/Na_2_HPO_4_ to a final concentration of 2.6 mmol/*L phosphate* with 3% FBS, with or without IL-1β (BioLegend) for 14 d.

### 2.7 Intracellular calcium content

For quantification of calcium content, calcium released into the supernatant was determined using the o-cresolphthalein complexone method as previously described [26], using the Calcium Colorimetric Assay (ab102505; Abcam, Cambridge, UK) and normalized to total protein content.

### 2.8 Alkaline phosphatase activity (ALP) assay

ALP activity of VSMCs was measured using LabAssay ALP (SensoLyte pNPP Alkaline Phosphatase Assay Kit. AS-72146; AnaSpec, Fremont, CA, USA), according to the manufacturer’s protocol. ALP activity was normalized to total protein content.

### 2.9 In vitro activation of bone marrow-derived macrophages (BMDMs)

Total BM cells were obtained from femurs and tibias of 8-week-old mice and cultured in a complete medium [DMEM supplemented with 10% fetal bovine serum (FBS), 1% penicillin/streptomycin, and 30 ng/mL macrophage-colony stimulating factor-1]. Non-adherent cells were collected after 24 h and differentiated for 7 d. For metabolic activation, differentiated BMDMs were primed with 200 ng/ml lipopolysaccharide (LPS, Sigma-Aldrich) for 3 h and polarized with 400 μM palmitate (PA, Sigma-Aldrich)-bovine serum albumin (BSA), 10 nM insulin and 30mM glucose for 3 h [27].

### 2.10 Enzyme-linked immunosorbent assay (ELISA)

Blood was collected in tubes from the left ventricle. Serum was separated by centrifugation at 2,000g for 20 min and stored at - 80 °C. The concentrations of IL-1β in serum were estimated using an ELISA kits (Mouse IL-1 beta/IL-1F2 Quantikine ELISA Kit, MLB00C; R&D Systems, Minneapolis, MN, USA) according to the manufacturer’s instructions.

### 2.11 Statistical analysis

Data were expressed as mean ± standard error of the mean (SEM). Mean values were compared using Student’s t-test or analysis of variance (ANOVA) followed by the Tukey-Kramer test to analyze intergroup differences. Significant differences among groups for dependent variables were detected using two-way ANOVA: male offspring vs. female offspring, phosphate treatment vs. phosphate plus IL-1β treatment. All analyses were performed using GraphPad Prism 8.3.0 Mac (GraphPad Software, LLC, San Diego, CA, USA).

## 3 Results

### 3.1 Maternal HFD increases calcified atherosclerotic plaque formation in male offspring

**Figure 1:**
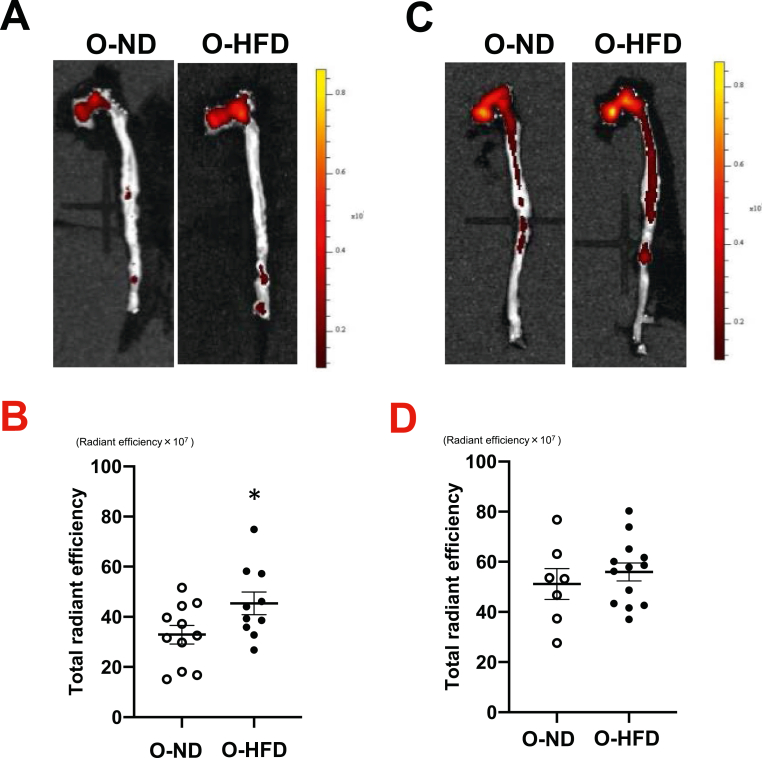
Maternal HFD exaggerates osteogenic activity in male offspring. (A and B) Representative photographs and quantitative measurements of fluorescent intensity of osteogenic activity in male offspring after 3 months of HCD feeding. Values represent mean ± SEM for 11 O-ND and 10 O-HFD mice. ∗*p* = 0.046 vs. O-ND. HCD, high-cholesterol diet; HFD, high-fat diet; ND, normal diet; O-ND, offspring of ND-fed dam; O-HFD, offspring of HFD-fed dam. (C and D) Representative photographs and quantitative measurements of fluorescent intensity of osteogenic activity in female offspring after 3 months of HCD feeding. Values represent mean ± SEM for 7 O-ND and 13 O-HFD mice. *p* = 0.483 vs. O-ND. HCD, high-cholesterol diet; HFD, high-fat diet; ND, normal diet; O-ND, offspring of ND-fed dam; O-HFD, offspring of HFD-fed dam.

**Figure 2:**
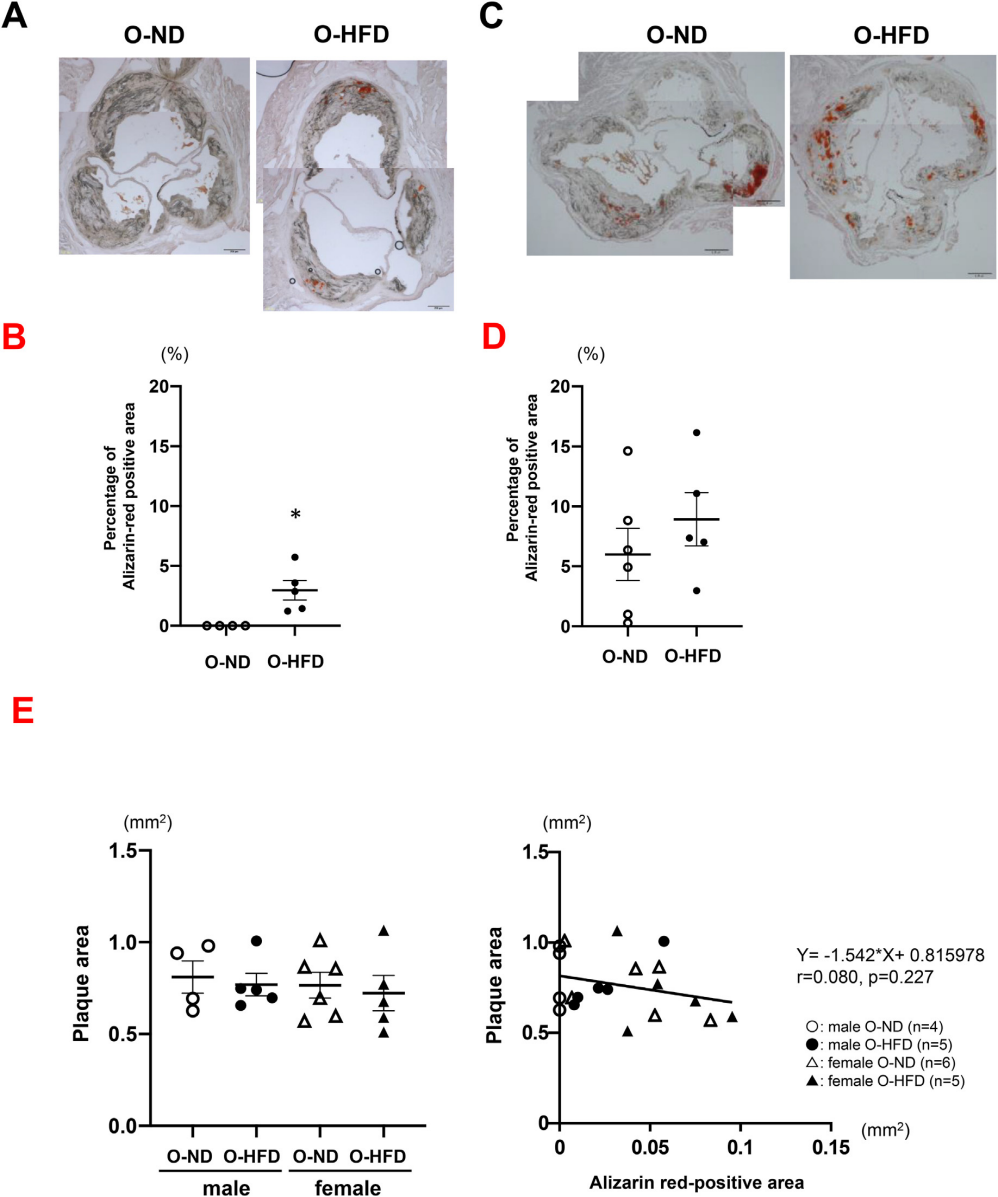
Maternal HFD increases alizarin-red-positive area in male offspring. (A and B) Representative photographs and quantitative measurements of alizarin-red-positive area in male offspring after 6 months of HCD feeding. Values represent mean ± SEM for 4 O-ND and 5 O-HFD mice. ∗*p* = 0.015 vs. O-ND. Scale bar shows 200 μm. HCD, high-cholesterol diet; HFD, high-fat diet; ND, normal diet; O-ND, offspring of ND-fed dam; O-HFD, offspring of HFD-fed dam. (C and D) Representative photographs and quantitative measurements of alizarin-red-positive area in female offspring after 6 months of HCD feeding. Values represent mean ± SEM for 6 O-ND and 5 O-HFD mice. *p* = 0.373 vs. O-ND. Scale bar represents 200 μm. HCD, high-cholesterol diet; HFD, high-fat diet; ND, normal diet; O-ND, offspring of ND-fed dam; O-HFD, offspring of HFD-fed dam. (E) Two-way ANOVA of plaque area (male vs. female) and correlation of plaque area with alizarin-red-positive area in offspring after 6 months of HCD feeding. HCD, high-cholesterol diet; HFD, high-fat diet; ND, normal diet; O-ND, offspring of ND-fed dam; O-HFD, offspring of HFD-fed dam.

After 3 months of HCD feeding, the osteogenic activity of the thoracic aorta was assessed using an ex vivo imaging system. Fluorescent intensity was significantly higher in male O-HFD as compared to those of O-ND (Figures 1A and 1B). In contrast, there was no significant difference between the female O-HFD and O-ND (Figures 1C and 1D). We next examined the alizarin red-positive area in the aortic root. However, alizarin-red-positive areas were scarcely observed in both groups of male mice fed an HCD for 3 months (Supplementary Figure 2). Therefore, we examined the alizarin red-positive area in HCD-fed mice for 6 months and found that it was significantly larger in male O-HFD than in male O-ND (Figures 2A and 2B), whereas it was slightly, but not significantly, larger in female O-HFD than in female O-ND (Figures 2C and 2D). We also examined the sex differences by using 2-way analysis of variance. The osteogenic activity was significantly greater in female offspring rather than in male offspring (p-interaction by sex = 0.001, p-interaction by maternal diet = 0.050). Likewise, plaque calcification assessed by alizarin-red-positive areas was significantly greater in female offspring (p-interaction by sex = 0.004, p-interaction by maternal diet = 0.121). Although there was no difference between female O-ND and O-HFD, osteogenic activity and atherosclerosis-related calcification were greater in female offspring than in male offspring.

To investigate whether higher alizarin red-positive area in female offspring is related with the plaque development, we examined the sex differences in plaque area using 2-way analysis of variance. The plaque area was not greater in female offspring than in male offspring (p-interaction by sex = 0.580, p-interaction by maternal diet = 0.610) (Figure 2E). Furthermore, alizarin red-positive area was not significantly correlated with plaque area in offspring (r = 0.080, p = 0.227) (Figure 2E). These findings suggest that augmented vascular calcification in female offspring is not responsible for the plaque progression.

### 3.2 Maternal HFD-associated atherogenic calcification is independent of plaque progression

**Figure 3:**
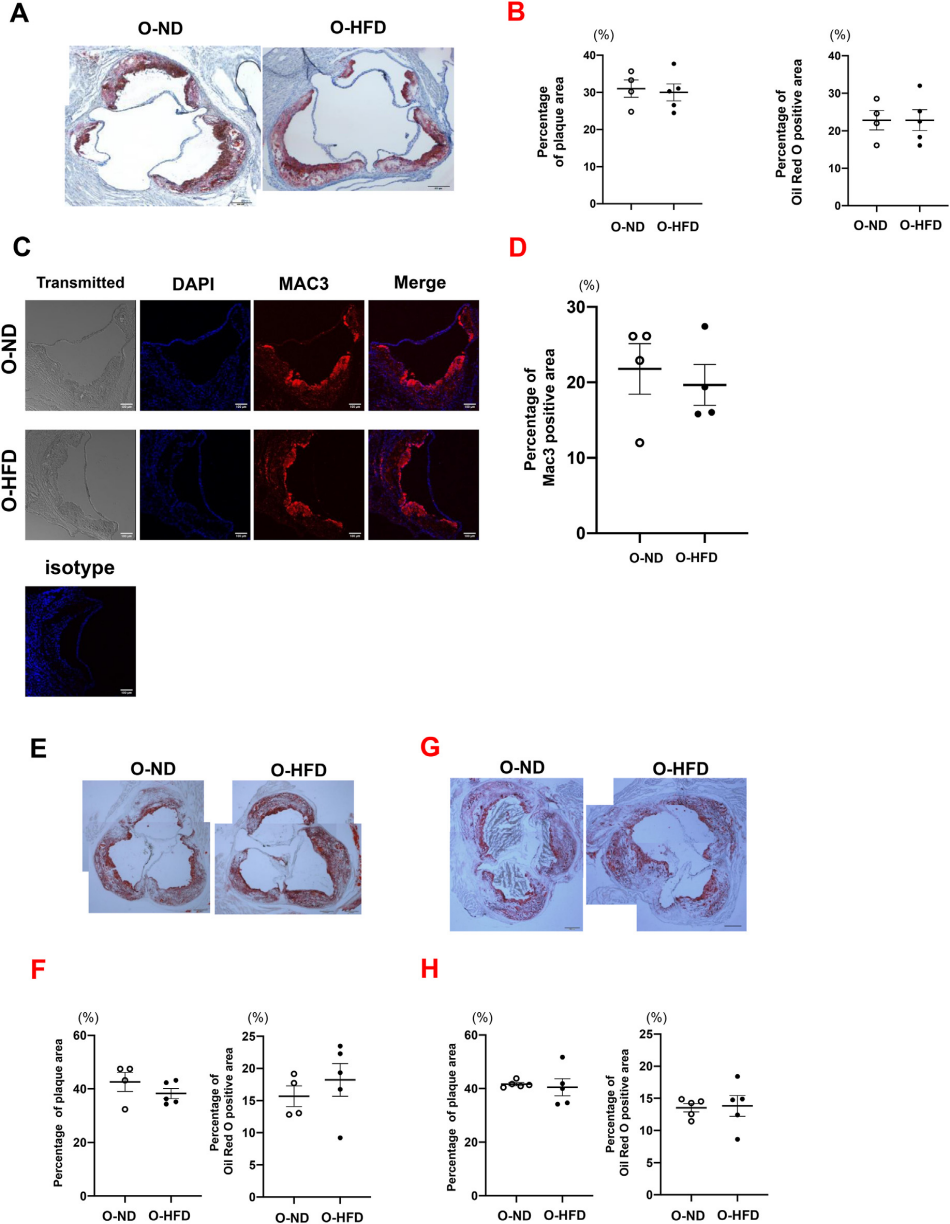
Maternal diet does not affect plaque size and macrophage accumulation. (A and B) Representative images of an oil-red O-stained aortic roots and quantitative analysis of percent plaque area and oil-red O-positive area in 3 months of HCD-fed male mice. Values are mean ± SEM for 4 (O-ND) and 5 (O-HFD). *p* = 0.773 and 0.999 vs. O-ND, respectively. Scale bar shows 200 μm. HCD, high-cholesterol diet; HFD, high-fat diet; ND, normal diet; O-ND, offspring of ND-fed dam; O-HFD, offspring of HFD-fed dam. (C and D) Representative confocal immunofluorescence images of Mac-3-stained aortic root and quantitative analysis of Mac-3-positive area. Values are mean ± SEM for 4 (O-ND) and 4 (O-HFD). *p* = 0.640 vs. O-ND. Scale bar represents 100 μm. HFD, high-fat diet; ND, normal diet; O-ND, offspring of ND-fed dam; O-HFD, offspring of HFD-fed dam. (E and F) Representative images of an oil-red O-stained aortic roots and quantitative analysis of percent plaque area and oil-red O-positive area in 6 months of HCD-fed male mice. Values are mean ± SEM for 4 (O-ND) and 5 (O-HFD). *p* = 0.487 and 0.450 vs. O-ND, respectively. Scale bar shows 200 μm. HCD, high-cholesterol diet; HFD, high-fat diet; ND, normal diet; O-ND, offspring of ND-fed dam; O-HFD, offspring of HFD-fed dam. (G and H) Representative images of an oil-red O-stained aortic roots and quantitative analysis of percent plaque area and oil-red O-positive area in 6 months of HCD-fed female mice. Values are mean ± SEM for 5 (O-ND) and 5 (O-HFD). *p* = 0.487 and 0.450 vs. O-ND, respectively. Scale bar shows 200 μm. HCD, high-cholesterol diet; HFD, high-fat diet; ND, normal diet; O-ND, offspring of ND-fed dam; O-HFD, offspring of HFD-fed dam.

To examine whether augmented calcification of male O-HFD is dependent on plaque progression, the percentages of plaque and oil-red O-positive areas were analyzed. There was no difference between the two groups of male mice fed HCD for 3 months (Figures 3A and 3B). Furthermore, the MAC-3-positive area was similar for the two groups of 3 months HCD-fed male mice (Figures 3C and 3D), suggesting that plaque size and macrophage accumulation are not associated with the increased calcification in male O-HFD. Blood pressure and heart rate before HCD feeding were equivalent between the two groups (Supplementary Figure 3). The lipid profile after HCD feeding for 3 months showed no difference between the two groups (Supplementary Figure 4). These findings show that augmented calcified plaque formation in male O-HFD is independent of hemodynamic variables and lipid profiles. We further analyzed plaque characteristics after 6 months of HCD feeding. There was no difference between O-ND and O-HFD in both gender of offspring (Figures 3E, 3F, 3G, and 3H). Two-way analysis of variance showed that the percentages of plaque and oil-red O-positive areas were not different between male and female (p-interaction by sex = 0.807 and 0.840, p-interaction by maternal diet = 0.285 and 0.436, respectively).

### 3.3 Maternal HFD augments phosphate-induced VSMCs transformation to osteochondrocytic-like phenotype in combination with IL-1β treatment

**Figure 4:**
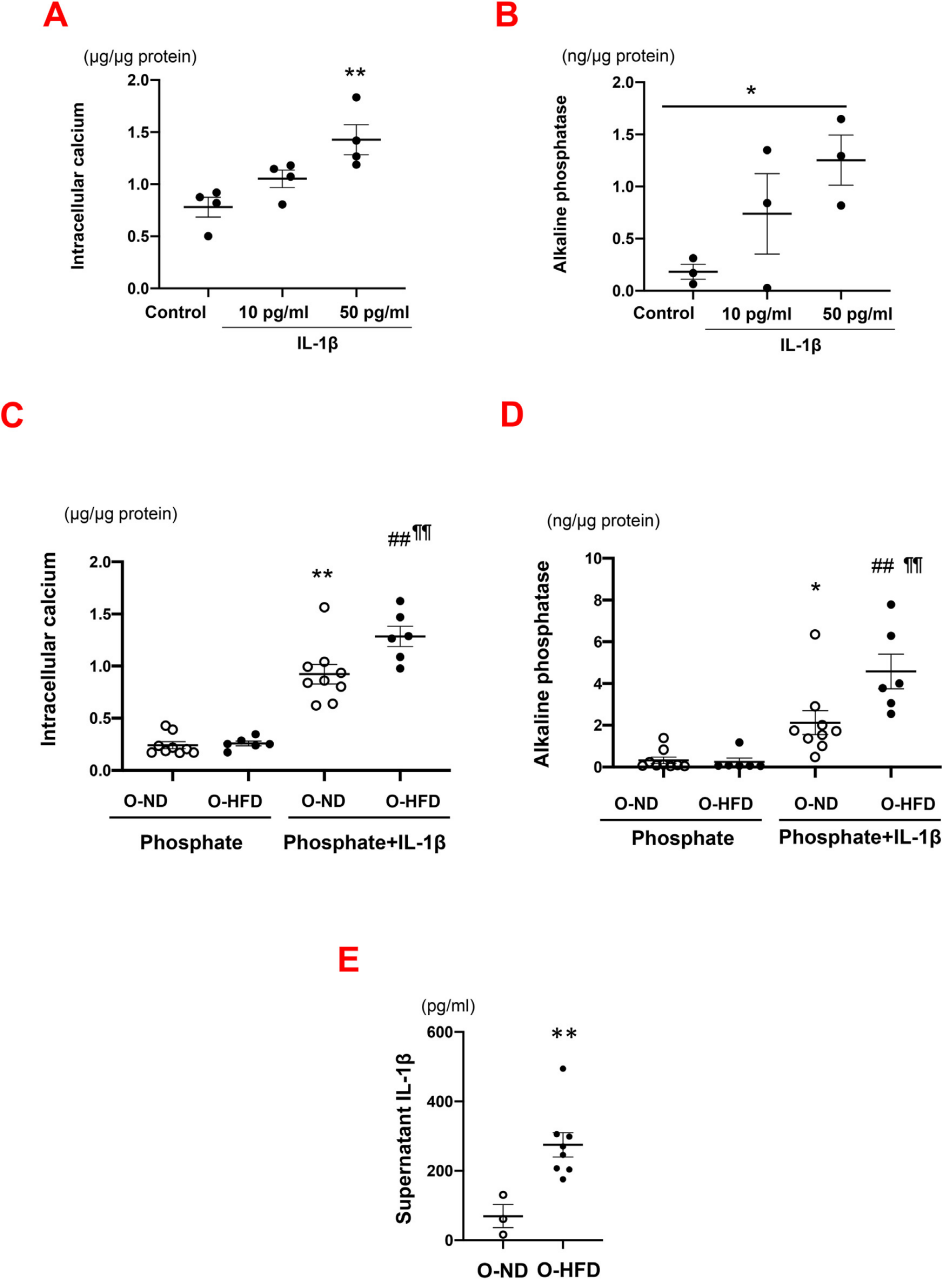
Maternal HFD increases phosphate plus IL-1β-induced transformation of VSMCs to osteochondrocytic-like phenotype. (A and B) Calcium concentration and ALP activity in VSMCs of wild-type mice after phosphate plus IL-1β stimulation. Values represent mean ± SEM for 4 in each group. ∗*p* = 0.030, ∗∗*p* = 0.007 vs. control. ALP, alkaline phosphatase activity; VSMCs, vascular smooth muscle cells; IL-1β, interleukin-1β. (C) Calcium concentration in VSMCs after phosphate plus IL-1β (10 pg/ml) stimulation. Values represent mean ± SEM for 9 O-ND and 6 O-HFD in each group. ∗∗*p* < 0.0001 vs. O-ND (phosphate). ^##^*p* < 0.0001 vs. O-HFD (phosphate). ^¶¶^*p* = 0.009 vs. O-ND (phosphate plus IL-1β). VSMCs, vascular smooth muscle cells; IL-1β, interleukin-1β; HFD, high-fat diet; ND, normal diet; O-ND, offspring of ND-fed dam; O-HFD, offspring of HFD-fed dam. (D) ALP activity in VSMCs after phosphate plus IL-1β (10 pg/ml) stimulation. Values represent mean ± SEM for 9 O-ND and 6 O-HFD in each group. ∗*p* = 0.041 vs. O-ND (phosphate). ^##^*p* < 0.0001 vs. O-HFD (phosphate). ^¶¶^*p* = 0.010 vs. O-ND (phosphate plus IL-1β). ALP, alkaline phosphatase activity; VSMCs, vascular smooth muscle cells; IL-1β, interleukin-1β; HFD, high-fat diet; ND, normal diet; O-ND, offspring of ND-fed dam; O-HFD, offspring of HFD-fed dam. (E) Supernatant concentration of IL-1β in metabolic activation of BMDMs. Values represent mean ± SEM for 3 O-ND, 8 O-HFD. ∗∗*p* = 0.009 vs. O-HFD. IL-1β, interleukin-1β; BMDMs, bone marrow-derived macrophages; HFD, high-fat diet; ND, normal diet; O-ND, offspring of ND-fed dam; O-HFD, offspring of HFD-fed dam.

We next examined the effect of maternal HFD on the transdifferentiation of VSMCs to osteochondrocytic-like cells using cultured VSMCs of the thoracic aorta. Osteochondrocytic-like cells differentiation was induced by treatment with phosphate, as previously described [25]. As phosphate-induced transformation to an osteochondrocytic-like phenotype is augmented by IL-1β treatment [19], we first examined the effect of IL-1β on VSMC transdifferentiation using wild-type mice. IL-1β treatment significantly increased calcium concentration and ALP activity in a dose-dependent manner (Figures 4A and 4B). Next, we compared the effect of phosphate plus IL-1β treatment on VSMC transdifferentiation between O-ND and O-HFD. Calcium concentration and ALP activity after phosphate stimulation were equivalent between the two groups. However, they were markedly increased after phosphate plus IL-1β stimulation, the extent of which was significantly higher in O-HFD (Figures 4C and 4D). These findings suggest that VSMCs in O-HFD are more likely to differentiate into osteochondrocytic-like cells in combination with IL-1β.

### 3.4 Maternal HFD increases IL-1β secretion from BMDMs after metabolic activation

It has been reported that inflammatory macrophages are a major source of IL-1β in atherosclerotic plaques [28], and we showed that BMDMs of wild-type HFD produced and released significantly higher IL-1β into the supernatant after metabolic activation, as compared to those in the wild-type O-ND [6]. Therefore, we examined IL-1β concentration in the supernatant after metabolic activation in apoE^−/−^ BMDMs. Consistent with our previous finding, IL-1β concentration was significantly higher in BMDMs from O-HFD than in BMDMs from O-ND (Figure 4E). These findings suggest that enhanced production of IL-1β in BMDMs and subsequent IL-1β signaling pathway in VSMCs could contribute to accelerated calcified atherosclerotic plaque formation in O-HFD.

## 4 Discussion

In this paper, we first showed that maternal HFD intake increased the development of atherogenic vascular calcification independent of plaque progression. Intracellular calcium content and ALP activity after phosphate plus IL-1β stimulation were markedly higher in the VSMCs of offspring of HFD-fed dams, suggesting that maternal HFD promotes VSMC transdifferentiation towards an osteochondrocytic-like phenotype along with macrophage-derived IL-1β. Our findings may provide new perspectives on the mechanisms of maternal HFD-associated CVD development with respect to cardiovascular calcification.

Cardiovascular calcification impairs the homeostasis of the cardiovascular system [12, 13]. Steitz et al. [15] first reported the phenotypic transition of VSMCs during spontaneous arterial calcification using matrix GLA protein (MGP) deficient mice. Speer et al. [16] showed that osteogenic transdifferentiation of VSMCs plays a critical role in the early stages of atherogenic vascular calcification using a lineage tracing approach. Lee et al. [17] have also demonstrated that TLR-2-mediated MAPK signaling promotes chondrogenic transdifferentiation of VSMCs, leading to augmented atherogenic calcification in apoE^−/−^ mice. Transdifferentiation of VSMCs to osteo/chondrogenic phenotype during calcification critically depends on environmental stresses such as inflammatory response and the surrounding extracellular matrix [14, 18, 21]. Ceneri et al. [19] showed that macrophage IL-1β promotes atherogenic calcification via Rac2-mediated osteogenic transcriptional programs in VSMCs. Consistently, we observed that maternal HFD did not affect intracellular calcium content and ALP activity in phosphate-stimulated VSMCs. However, combination with IL-1β treatment significantly augmented VSMC transdifferentiation. We also showed that BMDM-derived IL-1β expression in apoE−/− mice was markedly higher in O-HFD than in O-ND. Taken together, maternal HFD-associated vascular calcification is likely to be regulated by the interplay between VSMCs and inflammatory macrophages.

Hyperglycemia promotes VSMC transdifferentiation towards the osteogenic phenotype. Poetsch et al. [29] reported that hyperglycemic conditions promote osteogenic transdifferentiation of VSMCs by enhancing glucocorticoid-inducible kinase 1 (SGK1), in which nuclear translocation of SGK-1 activates NF-κB signaling pathways [30]. They also demonstrated that advanced glycation end products (AGEs) increased SGK1 expression in VSMCs [29]. In clinical studies, Raggi et al. [31] have shown that the average coronary calcium score was markedly higher in diabetic patients than in control subjects. Furthermore, Yamazoe et al. [32] reported that IR assessed by HOMA-IR was positively correlated with coronary artery calcium prevalence and progression. High fat/cholesterol diet intake can vary significantly from standard chow intake. However, consistent with our previously reported data [5, 6], body weight and cumulative caloric intake of offspring were not significantly different between O-ND and O-HFD. Given that maternal HFD exaggerates diet-induced IR along with hyperinsulinemic states [6], it is likely that maternal HFD-induced VSMC transdifferentiation is augmented by clinical complications such as metabolic disorders.

Vascular calcification and osteoporosis commonly coexist despite the calcium paradox phenomenon [33]. The mechanistic link between vascular calcification and osteoporosis has been intensively investigated. Demer et al. [34] showed that oxylipids promote the transdifferentiation of VSMCs to an osteogenic phenotype, while inhibiting osteoblast maturation via the nuclear factor-κB pathway. More recently, Mandatori et al. [35] reported that vitamin K2 prevented osteogenic transdifferentiation of resident VSMCs by augmenting MGP expression, while promoting osteoblast proliferation and its activity by enhancing osteocalcin expression. These findings suggest that maternal HFD is strongly associated with osteoporosis as well as cardiovascular calcification in the context of a reciprocal link between bone and vascular homeostasis. However, the effect of maternal HFD on osteoporosis has not yet been investigated, largely because osteoporosis and cardiovascular calcification have been recognized as age-related diseases. Recently, Chen et al. [36] reported that maternal obesity impairs osteodevelopment in offspring by augmenting osteoprogenitor senescence signaling via epigenetic programming. Shi et al. [37] also demonstrated that maternal diet-induced metabolic syndrome prevents the differentiation of osteoblast progenitors isolated from BM stromal cells, leading to osteopenia in offspring. Considering that osteoporosis is closely associated with incident CVD [ [38, 39]], the effect of maternal HFD on offspring osteopenia/osteoporosis is an emerging research area and need to be investigated in future studies.

Numerous clinical studies have shown that women have fewer calcified lesions and vessels of coronary arteries compared to men [40, 41]. These findings seem to be inconsistent with our findings that osteogenic activity and plaque calcification were significantly greater in female offspring rather than male offspring. However, Lee et al. recently reported that women had greater calcified plaque volume progression although women have lower calcium scores than men [42]. Likewise, Sato et al. have shown that in younger patients (<70), the prevalence of large calcification was significantly higher in women than in men [43]. Potential confounders such as smoking, diabetes mellitus, and hypertension are more prevalent in men, that may be associated with greater prevalence of coronary artery calcification. On the other hand, there are few animal experimental data addressing sex difference in vascular calcification by directly comparing between male and female animals. Augmented calcified plaque formation in female offspring and its underlying mechanisms need to be further investigated in future studies.

Our study shows that maternal HFD intake augments atherogenic calcification in adult offspring, independent of plaque progression. Moreover, in vitro VSMC transdifferentiation to osteochondrocytic-like cells was markedly accelerated by maternal HFD intake. These results provide novel insights into the underlying link between maternal HFD and CVD development in the context of cardiovascular calcification.

## Declarations

### Author contribution statement

Daisuke Miyawaki and Hiroyuki Yamada: Conceived and designed the experiments; Performed the experiments; Analyzed and interpreted the data; Wrote the paper.

Makoto Saburi, Naotoshi Wada, Shinichiro Motoyama and Hiroshi Kubota: Performed the experiments.

Noriyuki Wakana: Performed the experiments; Analyzed and interpreted the data; Wrote the paper.

Takeshi Sugimoto: Performed the experiments; Analyzed and interpreted the data.

Takehiro Ogata and Satoaki Matoba: Analyzed and interpreted the data; Wrote the paper.

Daisuke Kami: Contributed reagents, materials, analysis tools or data; Wrote the paper.

### Funding statement

Dr. Hiroyuki Yamada was supported by 10.13039/501100001691Japan Society for the Promotion of Science [JP15K09162].

### Data availability statement

Data included in article/supp. material/referenced in article.

### Declaration of interests statement

The authors declare no conflict of interest.

### Additional information

No additional information is available for this paper.

## Appendix A Supplementary data

Revised Supplementary Figure HELIYON-D-21-10659 Revised Supplementary Figure HELIYON-D-21-10659
