# Supplementary material for: Maternal high-fat diet promotes calcified atherosclerotic plaque formation in adult offspring by enhancing transformation of VSMCs to osteochondrocytic-like phenotype
Source: Heliyon. 2022 Sep 15;8(9):e10644. doi: 10.1016/j.heliyon.2022.e10644 (PMC9489965; doi:10.1016/j.heliyon.2022.e10644)
Supplement: Revised Supplementary Figure HELIYON-D-21-10659 [file mmc1.pptx]

## Slide 1
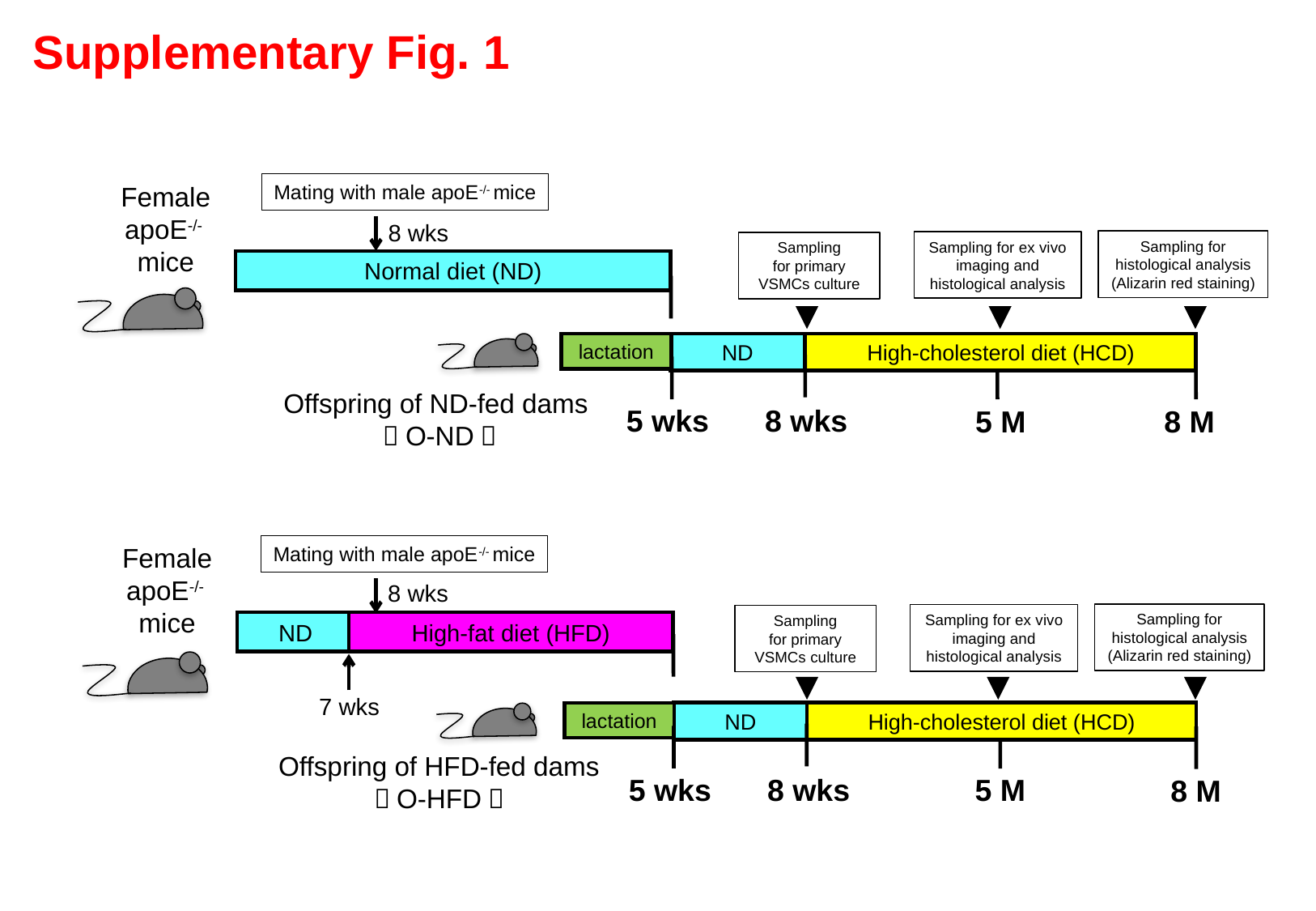

Supplementary Fig. 1
Mating with male apoE-/- mice
Female apoE-/- mice
8 wks
Sampling for histological analysis (Alizarin red staining)
Sampling for ex vivo imaging and histological analysis
Sampling
for primary VSMCs culture
Normal diet (ND)
lactation
ND
High-cholesterol diet (HCD)
Offspring of ND-fed dams
（O-ND）
5 wks
8 wks
8 M
5 M
Female apoE-/- mice
Mating with male apoE-/- mice
8 wks
Sampling for histological analysis (Alizarin red staining)
Sampling for ex vivo imaging and histological analysis
Sampling
for primary VSMCs culture
ND
High-fat diet (HFD)
7 wks
lactation
ND
High-cholesterol diet (HCD)
Offspring of HFD-fed dams
（O-HFD）
5 wks
8 wks
5 M
8 M

## Slide 2
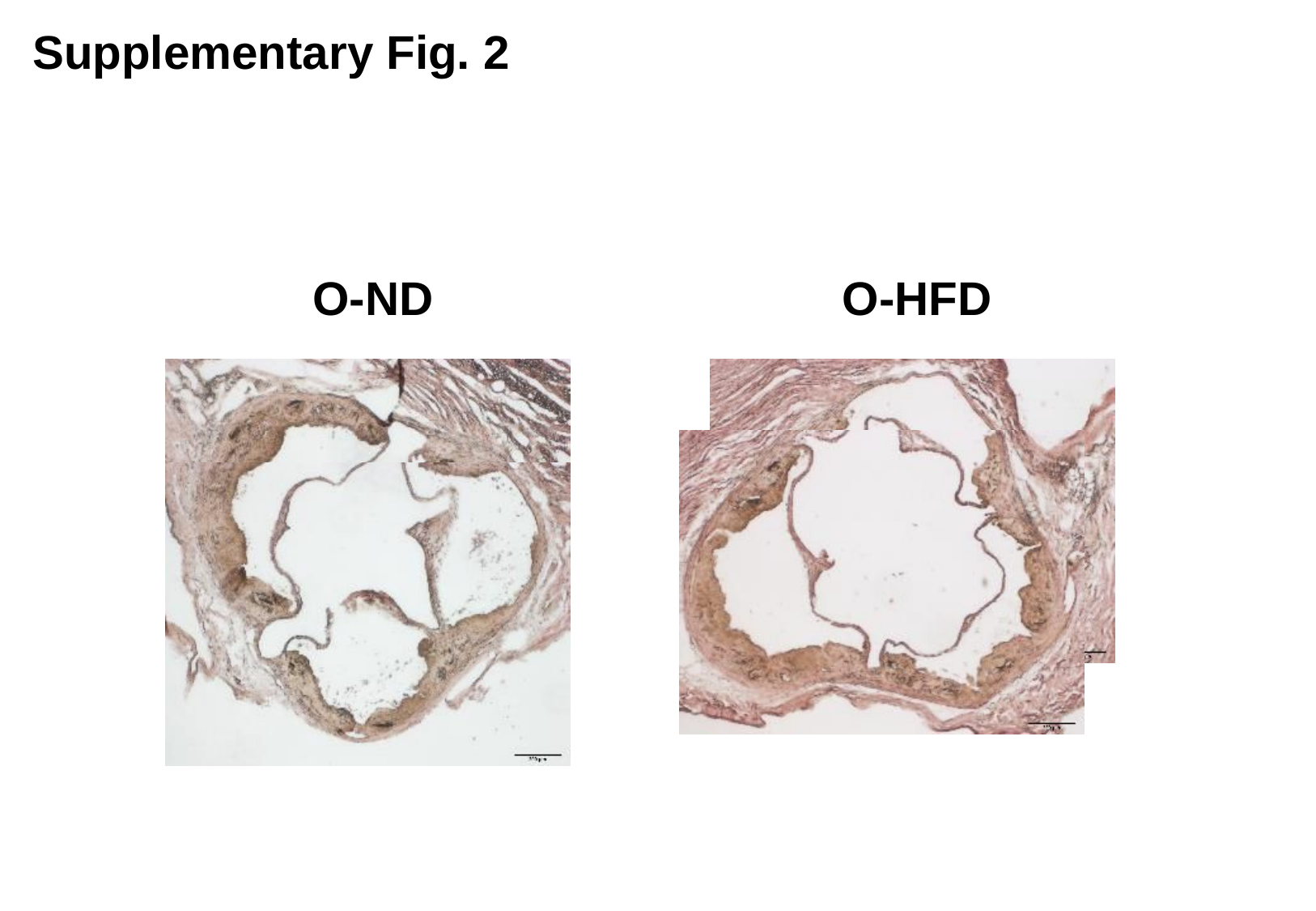

Supplementary Fig. 2
O-ND
O-HFD

## Slide 3
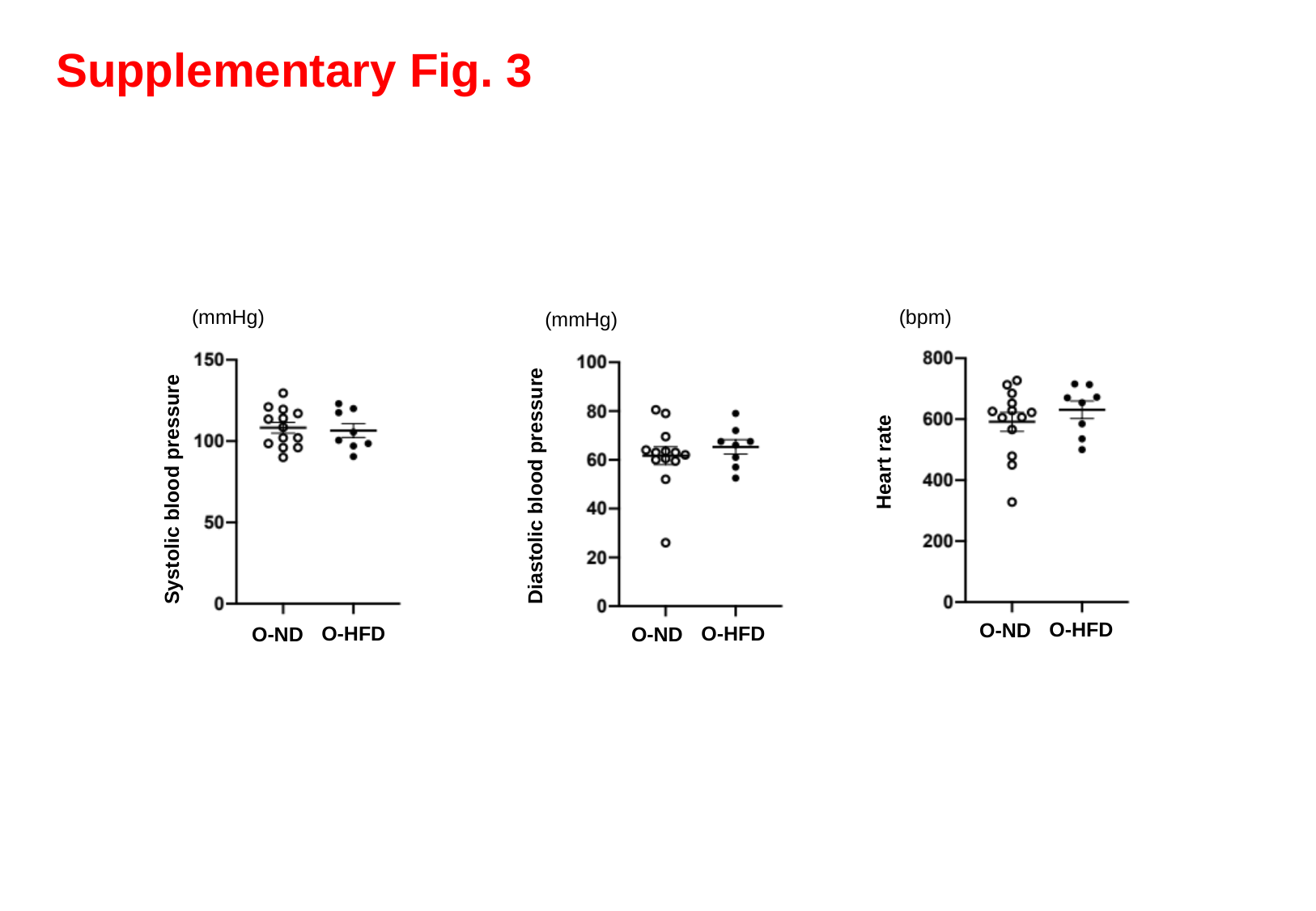

Supplementary Fig. 3
(bpm)
(mmHg)
(mmHg)
Heart rate
Diastolic blood pressure
Systolic blood pressure
O-HFD
O-ND
O-HFD
O-HFD
O-ND
O-ND

## Slide 4
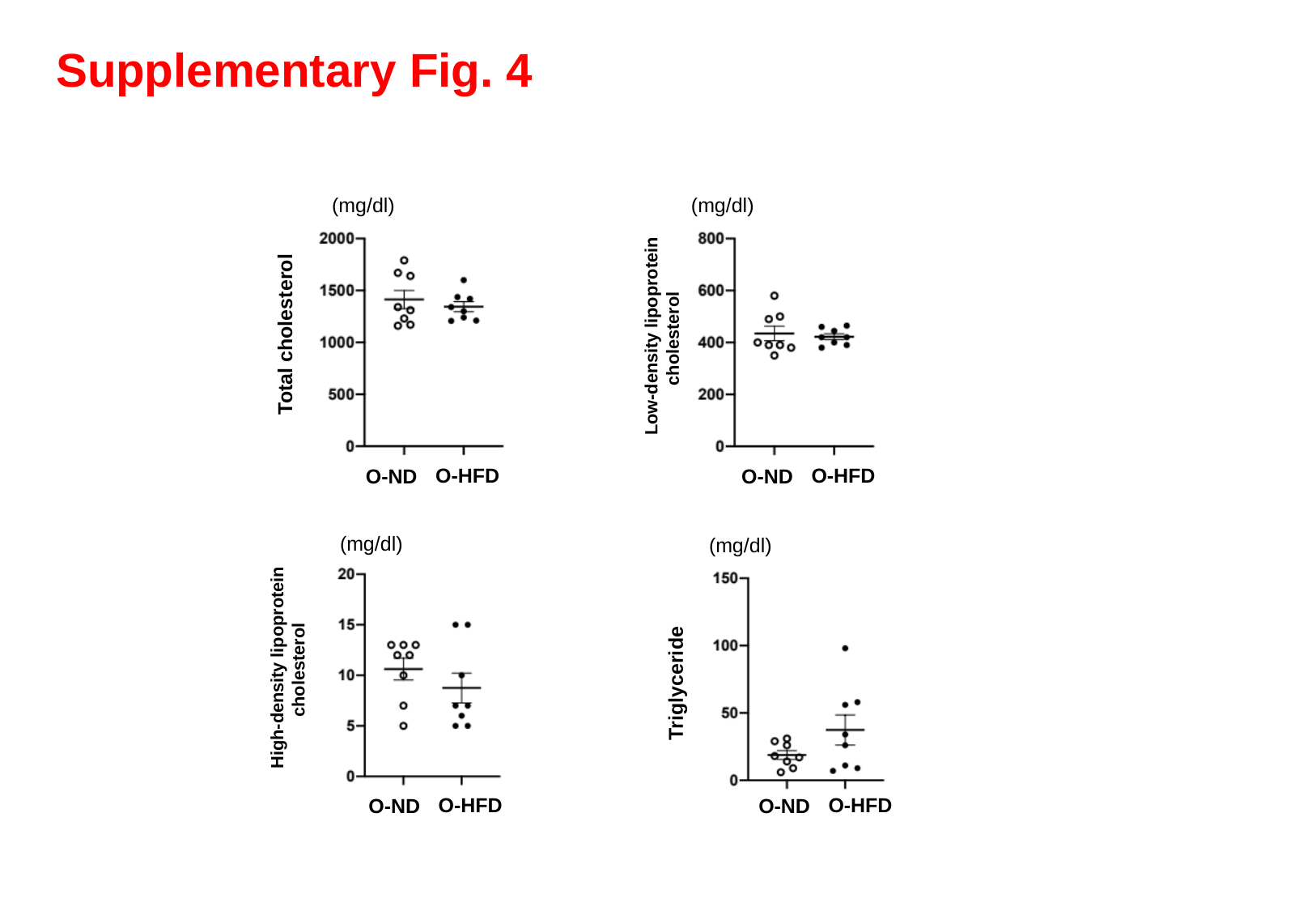

Supplementary Fig. 4
(mg/dl)
(mg/dl)
Low-density lipoprotein
cholesterol
Total cholesterol
O-HFD
O-HFD
O-ND
O-ND
(mg/dl)
(mg/dl)
High-density lipoprotein
cholesterol
Triglyceride
O-HFD
O-HFD
O-ND
O-ND
